# Investigation of the Usefulness of Hanger and Retention Bag and Double Suture Hanger Technique in Preventing Tumor Spillage During Gynecologic Laparoscopic Surgery for Mature Cystic Teratoma

**DOI:** 10.7759/cureus.77842

**Published:** 2025-01-22

**Authors:** Yu Horibe, Toshiyuki Kanno, Akira Nakabayashi, Jun Kumakiri, Tsutomu Tabata

**Affiliations:** 1 Obstetrics and Gynecology, Tokyo Women's Medical University, Tokyo, JPN

**Keywords:** dermoid cyst, double suture hanger technique, hanger and retention bag, laparoscopic cystectomy, teratoma

## Abstract

Since the laparoscopic surgical technique and quality for ovarian cystectomy largely depend on the primary surgeon, we investigated the usefulness and feasibility of the hanger and retention bag (HRB) and double suture hanger technique (DSHT) as surgical maneuvers to prevent ovarian tumor spillage, minimizing reliance on the surgeon’s skill to the greatest extent possible.

HRB and DSHT present several advantages. First, requiring only a retrieval bag and suture, HRB and DSHT are feasible, simple, and cost-effective, and can be performed at any facility with endoscopic surgical equipment. Additionally, the learning curve is extremely short. Second, the port from which the thread is pulled can be positioned anywhere, allowing for parallel, diamond, or other anomalous port configurations. Third, these techniques reduce the risk of tumor spillage during cystectomy. The cyst can be lifted upward toward the abdominal wall while being stored in the HRB, ensuring that even if the contents collapse, they are reliably trapped in the HRB by gravity. Even if the cyst is not fully retracted, fine adjustments can be made as needed using flexible traction. Fourth, while the absence of adhesions around the ovarian cyst is a prerequisite, this approach can also be applied to conditions other than mature cystic teratoma.

Furthermore, the HRB and DSHT described here can be used in combination with other surgical devices, depending on the surgeon’s skill. This suggests that they may contribute to the advancement of minimally invasive surgery and the prevention of complications. We will report the results of this study, along with the accumulation of cases, in the future.

## Introduction

Laparoscopic surgery is now widely accepted as a universal technique for treating benign ovarian tumors. Conversely, reports indicate that in approximately 50% of benign tumors, especially mature cystic teratoma, there is leakage of contents during dissection of the cyst. This leakage can cause peritonitis due to a chemical reaction, albeit at a very low frequency, leading to prolonged hospital stays and a decline in quality of life [1,2]. Malignant tumors also pose a problem, although with an incidence of approximately 1% [3]. Since surgical technique and quality depend heavily on the primary surgeon, we investigated the usefulness and feasibility of the hanger and retention bag (HRB) and double suture hanger technique (DSHT) as surgical maneuvers to prevent tumor spillage that do not depend, to the greatest extent possible, on the surgeon's skill.

## Technical report

**Figure 1:**
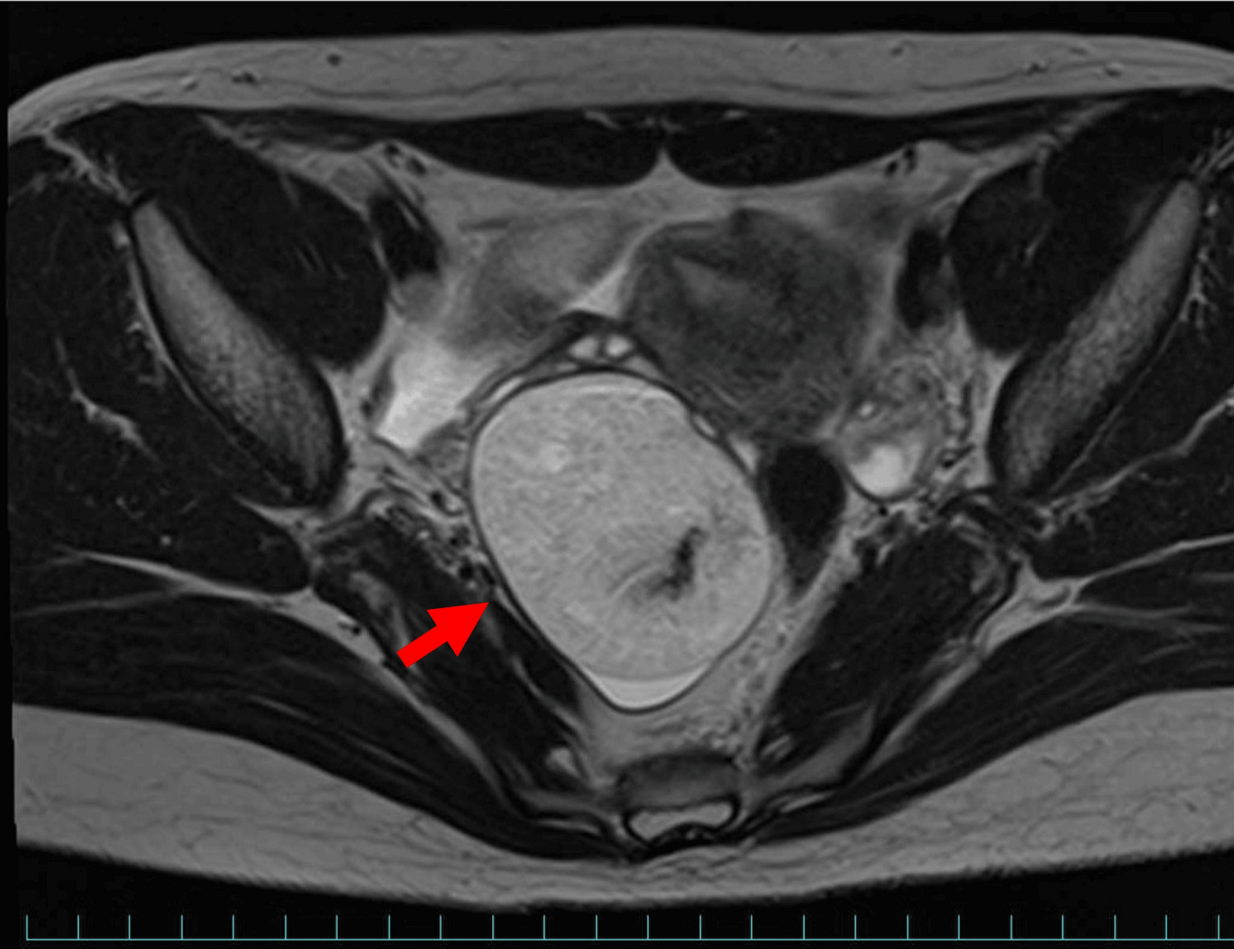
MRI of mature cystic teratoma on the right ovary. The red arrow indicates a 6 cm mature cystic teratoma on the right ovary.

The surgical technique of laparoscopic ovarian cystectomy for mature cystic teratoma was reviewed. The patient was 30 years old with no prior pregnancies or deliveries. There were no complications. Preoperative MRI revealed a suspected 6 cm mature cystic teratoma on the right ovary, prompting a laparoscopic right ovarian cystectomy (Figure 1).

The operation was performed with the patient in the open-leg position using parallel port placement (umbilical port 5 mm, left upward lateral abdominal port 12 mm, left downward lateral abdominal port 5 mm, and right assistant port 5 mm). Disposable monopolar, incision, and grasping forceps were utilized for the procedure. Additionally, the pouch of the LiNA EasyBag™ was employed as the retrieval bag for laparoscopic cystectomy.

**Figure 2:**
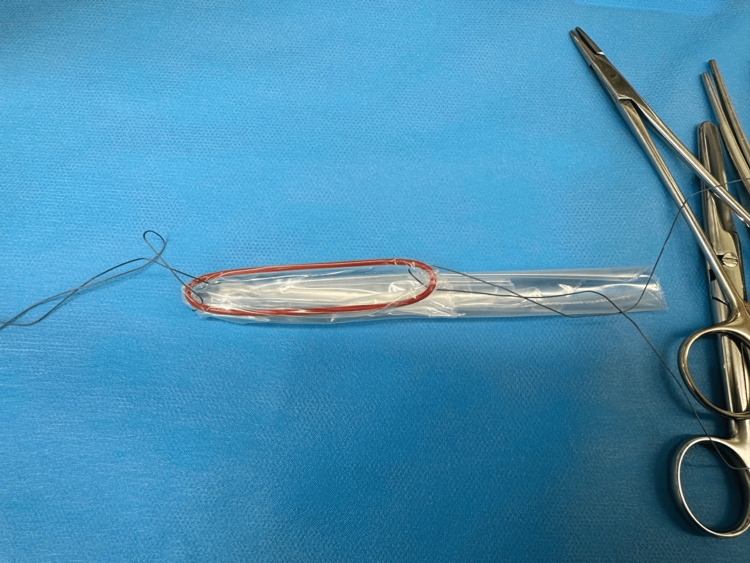
Hanger and retention bag (HRB) made of absorbable sutures and a retrieval bag.

The retrieval technique is described below. First, an HRB for storing the ovarian tumor was prepared preoperatively, as shown in Figure 2.

**Figure 3:**
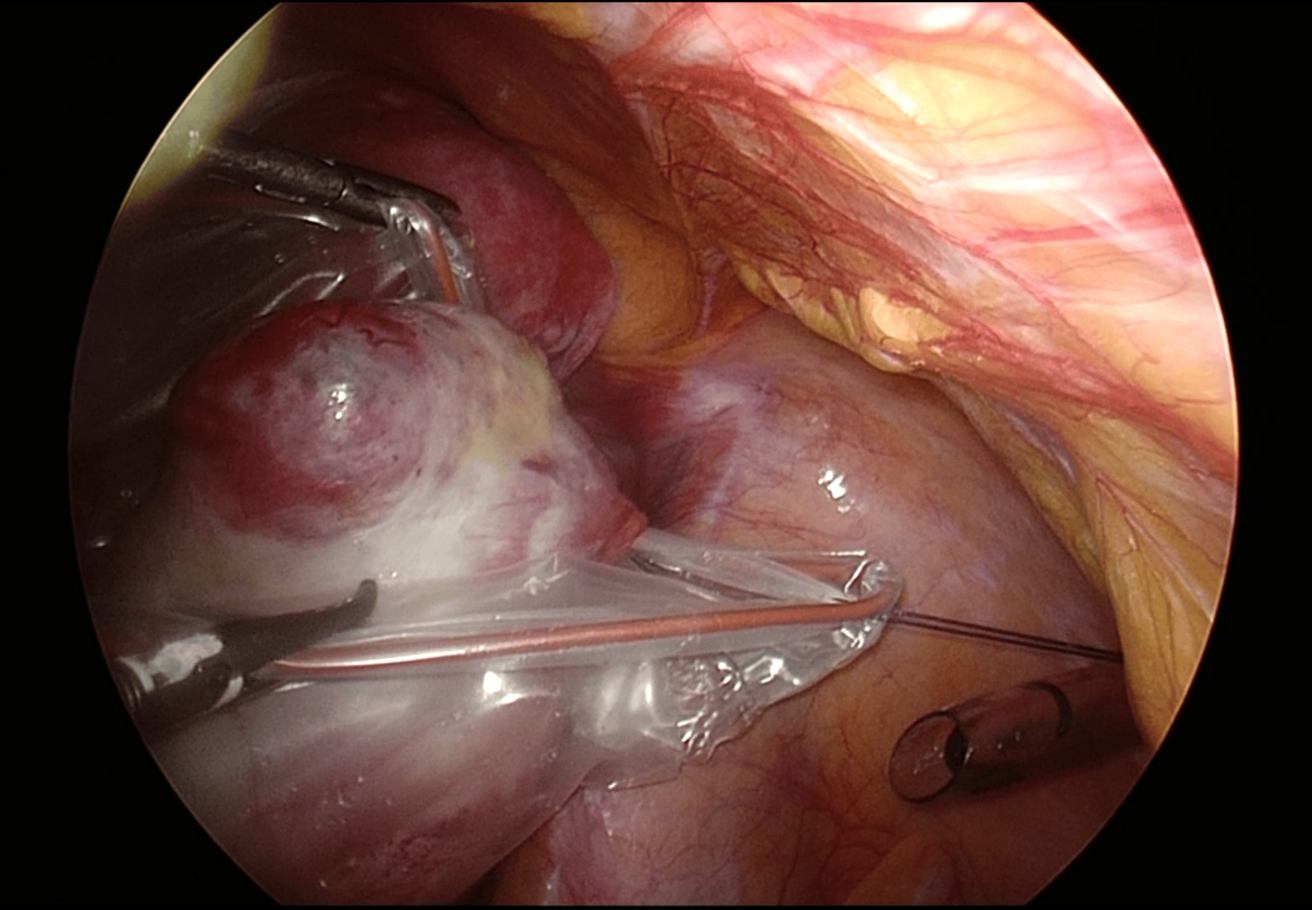
Image of fixed sutures. The sutures attached to the edge of the bag are pulled up through the left and right 5-mm ports.

The HRB can be made by any manufacturer and can be of any type, as long as it is appropriate for the size of the tumor. A 20-25 cm piece of suture was sewn around the top edge of the bag on both sides using ETHICON VICRYL 2-0™. The length may be adjusted for extremely obese patients or in cases of emaciation. Upon reaching the intraperitoneal space, the surgeon confirmed that there was no adhesion of the ovarian tumor and inserted the prepared bag through the 12-mm port into the Douglas fossa. Next, the ovarian tumor was placed into the bag, and the left margin suture was pulled up through the left surgeon's 5-mm port. The thread was grasped from outside the body with grasping forceps to prevent it from falling back into the intraperitoneal space. The right margin suture was also pulled up through the assistant's port and secured outside the body in the same manner (Figure 3).

**Figure 4:**
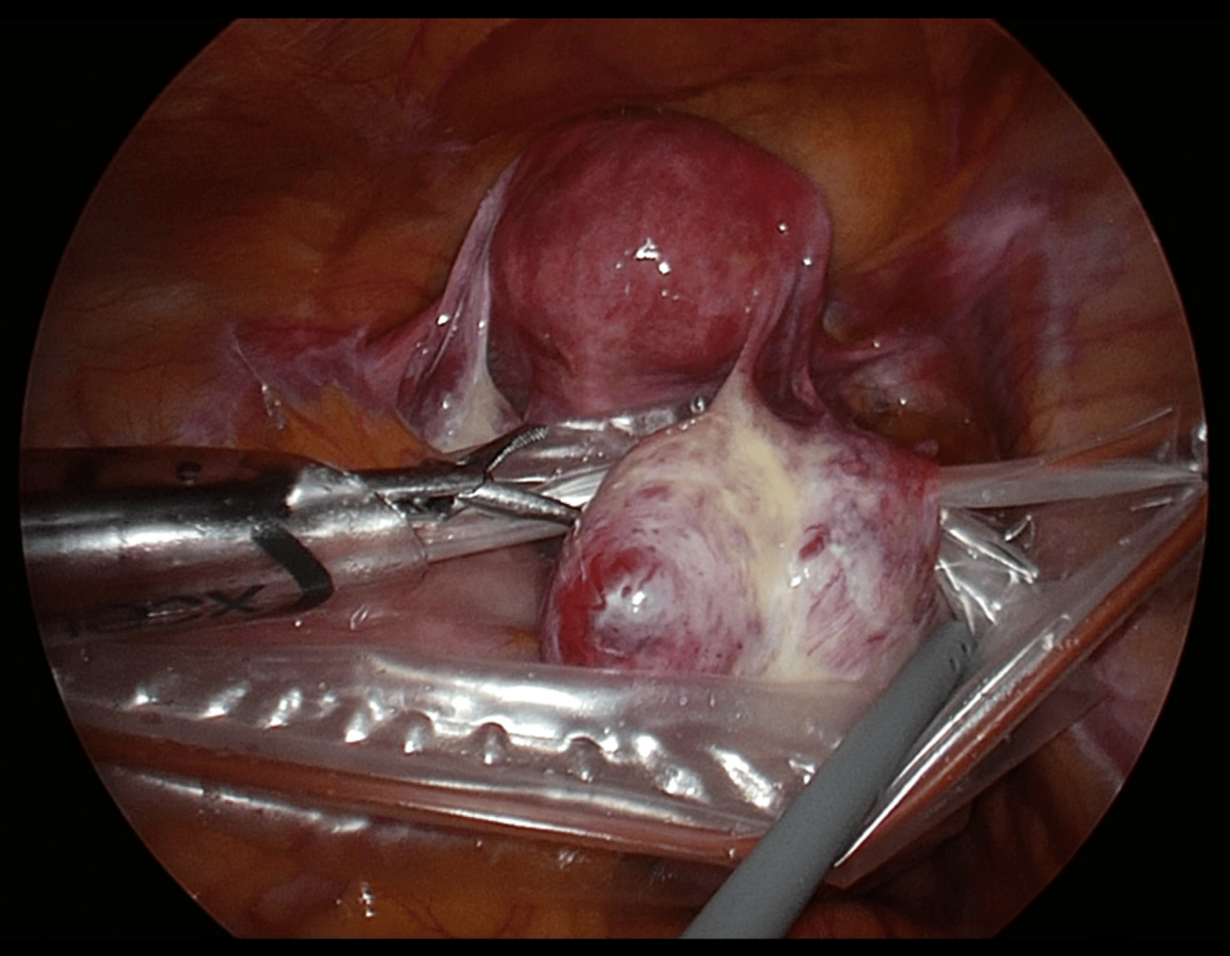
Ovarian cyst stored in HRB. The HRB is secured from both sides, providing sufficient space for manipulation during cyst removal. The sutures are pulled and secured from the pelvic floor to the abdominal wall, ensuring they remain centered between the pelvic floor and abdominal wall. HRB: Hanger and retention bag.

The bag was secured from both sides to allow enough space for the cystectomy, and both sutures were pulled until the bag stayed centered from the pelvic floor to the abdominal wall (Figure 4).

**Figure 5:**
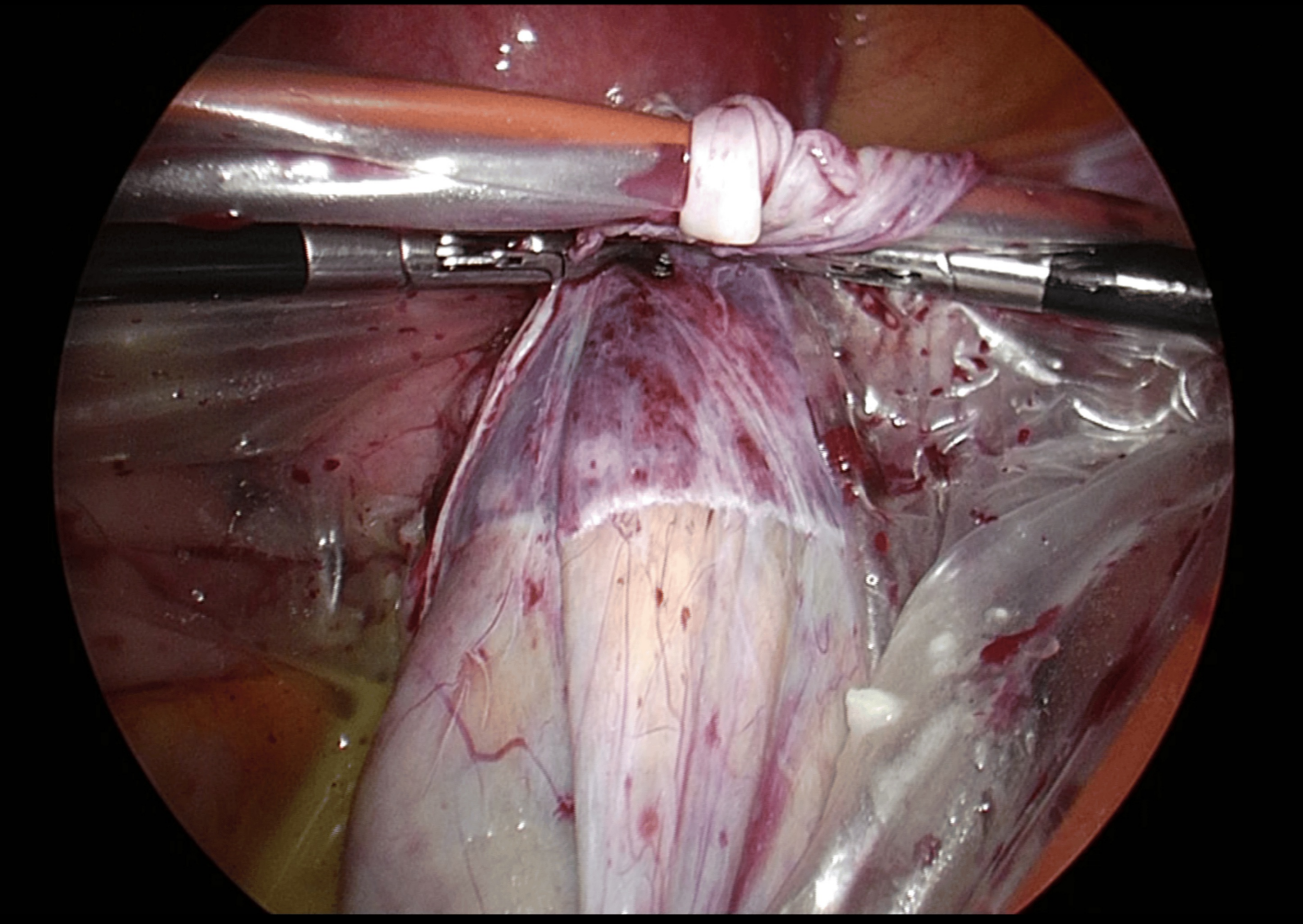
Image of cystectomy into HRB. The HRB is suspended from the right and left ports, allowing the dissection operation to be completed without leakage of contents outside the abdominal cavity. Any tumor spillage drips into the bag under the influence of gravity. HRB: Hanger and retention bag.

Even if contents leaked during the dissection, gravity kept the contents at the bottom of the bag, and the surgical operation did not invert the bag, preventing contents from flowing back into the abdominal cavity (Figure 5).

**Figure 6:**
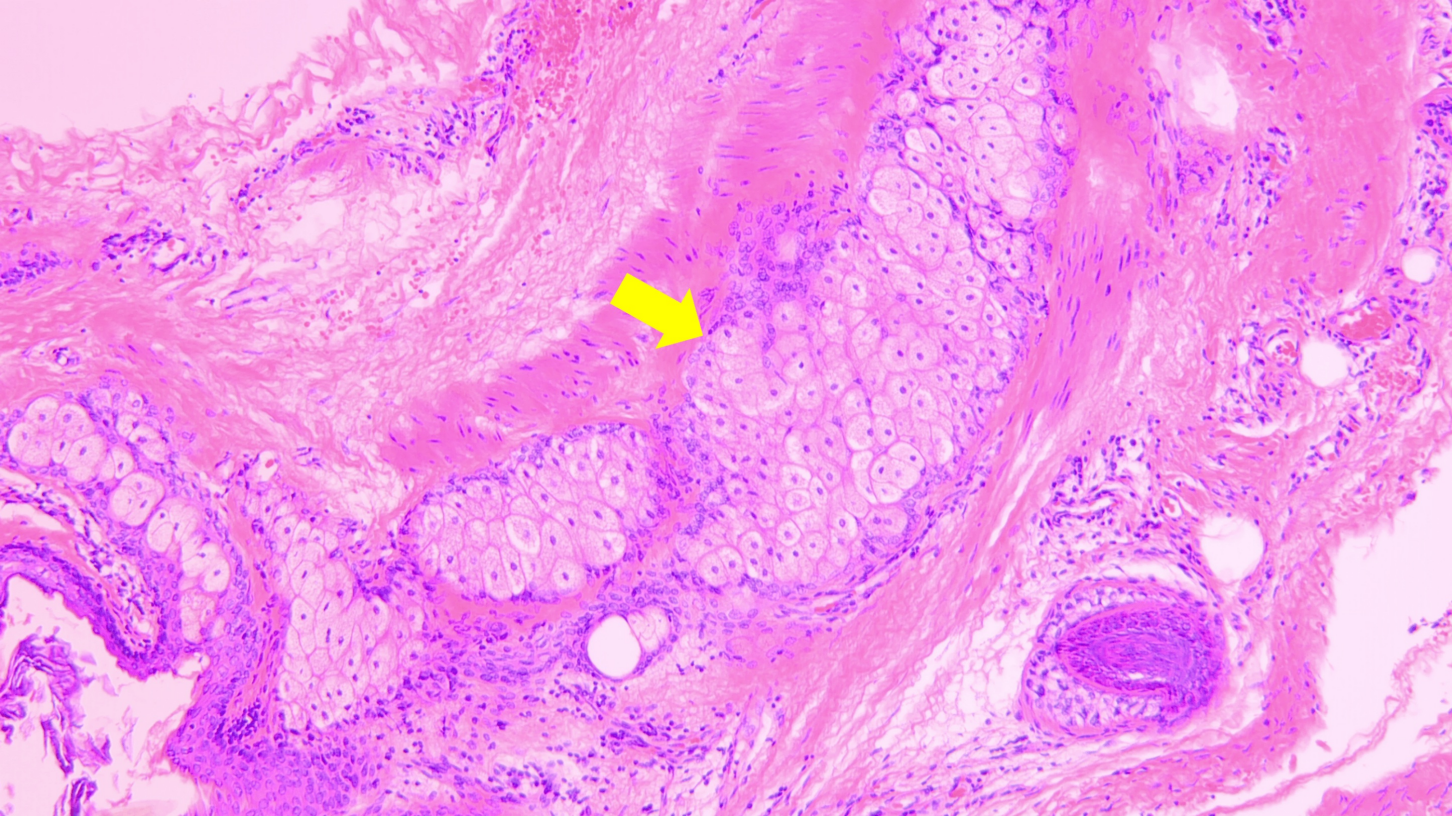
Microscopic findings. The slide includes adipose tissue (indicated by the yellow arrow) with no malignant findings observed.

After enucleation of the cyst, the right and left sutures were unclasped and withdrawn from the 5-mm ports gently. The surgeon then grasped the bag through the 12-mm port with grasping forceps and pulled it out of the intraperitoneal space. The operative time was 64 minutes, and bleeding totaled 3 mL. Although the contents collapsed during cystectomy, all leaked material was successfully recovered in the bag. The postoperative pathology result confirmed a mature cystic teratoma, with no malignant findings (Figure 6).

## Discussion

Various techniques have been investigated to prevent intraperitoneal tumor spillage during laparoscopic ovarian cyst enucleation. For example, mesial incision for laparoscopic dermoid cystectomy [4] and hydrodissection [5] have been reported as useful, but both techniques are highly dependent on the skill of the surgeon, making their recommendation across centers challenging. There are also reports of surgical plans for each step, but the impact of the surgeon’s skill cannot be denied [6,7]. In-bag retrieval methods have also been reported [8,9]; however, without proper lifting, there are many cases of tumor leakage from the bag.

HRB and DSHT present several advantages. First, the HRB and DSHT, requiring only a retrieval bag and suture, are feasible, simple, and cost-effective, and can be performed at any facility with endoscopic surgical equipment. In addition, the learning curve is extremely short. Second, the port from which the thread is pulled can be positioned anywhere, allowing for parallel, diamond, or other anomalous port configurations. Third, the risk of tumor spillage due to cystectomy can be reduced compared to previously reported techniques. The cyst can be lifted upward toward the abdominal wall while being stored in the HRB by the appropriate traction of the surgeon and assistant, so that even if the contents collapse, they can be reliably trapped in the HRB by gravity. Even if the cyst is not fully retracted, fine adjustments can be made as needed using flexible traction. Fourth, while the absence of adhesions around the ovarian cyst is a prerequisite, this approach can also be applied to conditions other than mature cystic teratoma.

Conversely, there are also disadvantages. First, once the HRB is fixed at the appropriate position, the surgeon can complete the surgery alone but must collaborate with an assistant until proper traction is achieved. Second, there is an increase in operative time of about 10 minutes to confirm the proper position of the cystectomy. Third, it cannot be used in cases of intraperitoneal adhesions. In particular, when advanced adhesions are present, there are concerns about prolonged hospitalization and the need for antibiotic administration [10]. In such cases, we would not hesitate to shift to laparotomy, regardless of tumor leakage.

Fourth, if the tumor size is so large that the traction thread cannot be confirmed or the HRB is not large enough, it is not indicated and other alternative methods, such as extracorporeal aspiration, should be considered [11,12].

## Conclusions

In addition, the HRB and DSHT techniques described here can be used in combination with other surgical devices, depending on the surgeon’s skill. This suggests that they may contribute to the advancement of minimally invasive surgery and the prevention of complications. We will report the results of this study, along with the accumulation of cases, in the future.
